# There Are no Short-Term Longitudinal Associations Among Interoceptive Accuracy, External Body Orientation, and Body Image Dissatisfaction

**DOI:** 10.32872/cpe.v2i2.2701

**Published:** 2020-06-30

**Authors:** Raechel E. Drew, Eszter Ferentzi, Benedek T. Tihanyi, Ferenc Köteles

**Affiliations:** a Institute of Psychology, ELTE Eötvös Loránd University, Budapest, Hungary; b Centre for Infant Cognition, Department of Psychology, University of British Columbia, Vancouver, Canada; c Doctoral School of Psychology, ELTE Eötvös Loránd University, Budapest, Hungary; d Institute of Health Promotion and Sport Sciences, ELTE Eötvös Loránd University, Budapest, Hungary; Philipps-University of Marburg, Marburg, Germany

**Keywords:** interoceptive accuracy, body image, self objectification, body surveillance, public body consciousness, body image dissatisfaction

## Abstract

**Background:**

Objectification theory assumes that individuals with low level of interoceptive accuracy may develop an external orientation for information concerning their body. Past research has found associations between interoceptive accuracy and body image concerns. We aimed to explore temporal relationships between the tendency to monitor one's body from a third-party perspective, body image dissatisfaction, and interoceptive accuracy.

**Method:**

In a short longitudinal research, 38 Hungarian and 59 Norwegian university students completed the Schandry heartbeat tracking task and filled out baseline and follow-up questionnaires assessing private body consciousness, body surveillance, and body image dissatisfaction 8 weeks apart.

**Results:**

Interoceptive accuracy and indicators of external body orientation did not predict body image dissatisfaction after controlling for gender, nationality, and body image dissatisfaction at baseline. Similarly, body surveillance was not predicted by baseline levels of interoceptive accuracy and body image dissatisfaction.

**Conclusion:**

Contrary to the tenets of objectification theory, body image dissatisfaction and body surveillance are not predicted by interoceptive accuracy over a short period of time among young individuals.

## Abstract (highlights)

- Past research suggests that an individual's ability to detect their own internal signals may have important implications for body monitoring and body image.
- We did not find the expected temporal associations among interoceptive accuracy and body image-related variables.
- Culture and gender were predictors of body image dissatisfaction, an important consideration when designing interventions targeting body image concerns.

Interoception, the perception of sensations originating from within the body, is related to many aspects of daily functioning, including subjective emotional experience, decision making, and our sense of self (Craig, 2002; Damasio, 1999; Tsakiris, 2017). In the insular cortex, interoceptive and exteroceptive information converge, are processed and integrated, and provide us with a sense of the physiological status of our entire body, or a feeling of *embodiment* (Craig, 2015; Herbert & Pollatos, 2012; Tsakiris, 2017). Interoceptive accuracy (IAc) is the dimension of interoception that specifically describes accurately detecting one’s own bodily signals (Ceunen, Van Diest, & Vlaeyen, 2013; Garfinkel, Seth, Barrett, Suzuki, & Critchley, 2015). It is typically measured via behavioral test, as opposed to self-report. Individuals with low levels of IAc seem to have more difficulties maintaining a healthy body image and may be more likely to experience body dissatisfaction and eating disorders (Badoud & Tsakiris, 2017; Cash & Deagle, 1997; Herbert & Pollatos, 2012; Pollatos et al., 2008), although this is not always the case (Pollatos & Georgiou, 2016).

Body image as a concept refers to the mental representation of one's own body, but is multifaceted in that it includes perceptual, affective, and cognitive components (Badoud & Tsakiris, 2017; Cash & Pruzinsky, 1990, 2002; Gaudio & Quattrocchi, 2012; Tiggemann & Lynch, 2001). Past research has approached body image concerns from several different perspectives (i.e., body image dissatisfaction, internalized thin ideals); thus, body image has been widely used as an umbrella term for several related constructs (Badoud & Tsakiris, 2017). In light of an absence of a clear definition, Badoud and Tsakiris (2017, p. 7) have defined body image very simply as “the conscious, predominantly visual, mental representation of one’s own body and of our perceptual, cognitive and affective attitudes towards it”. It is considered the product of a complex aggregation of bottom up and top down information - signals originating from within and outside of the body (Craig, 2015; Eshkevari, Rieger, Longo, Haggard, & Treasure, 2012; Suzuki, Garfinkel, Critchley, & Seth, 2013). It is proposed that the balance between processing of interoceptive and exteroceptive cues is central to the stability and health of our body image (Badoud & Tsakiris, 2017; Tsakiris, 2017). Predictive coding models suggest that individuals who do not perceive interoceptive signals accurately may learn to rely more on external cues when assessing the body's status due to the imprecision of predictions (i.e., top-down representations) based on prior inaccuracies (Ainley, Apps, Fotopoulou, & Tsakiris, 2016). In line with this idea, Tsakiris and colleagues (2011) found that individuals with low IAc were more likely to assume ownership of a false body part, highlighting the level of disembodiment and body image distortion that can occur when accurate perception of internal signals is dampened. Objectification Theory (Fredrickson & Roberts, 1997) indicates that when the psychological experience of the body (i.e., embodiment) is predominantly informed by external sources of information, there will be greater exposure to negative cultural cues (i.e., unattainable beauty ideals, objectifying media imagery). This, in turn, contributes to discrepancies between the idealized body image and perceived actual appearance of the body, through further internalization of ideals and making salient any existing discrepancies (McKinley & Hyde, 1996). Furthermore, discrepancies between the perceived self and an internalised ideal self (i.e., evaluation), plus a high level of importance placed on matching that ideal (i.e., investment) can produce body image dissatisfaction (Cash, 2012; Cash & Pruzinsky, 2002). Concerning healthy young individuals, women with attenuated IAc exhibit higher levels of body image dissatisfaction (Emanuelsen, Drew, & Köteles, 2015). Similarly, Duschek and colleagues (2015) found that individuals with greater IAc had a more positive body image.

Self objectification is the acculturated tendency to view one's own body as an object, to evaluate it based on appearance rather than functionality, and to experience oneself from a third-party perspective (Ainley & Tsakiris, 2013; Calogero, Tantleff-Dunn, & Thompson, 2010; Fredrickson & Roberts, 1997). Habitual self-monitoring, an integral aspect of self objectification, is referred to in the literature as body surveillance (Calogero et al., 2010; Grippo & Hill, 2008; McKinley & Hyde, 1996). Body surveillance is accepted as the behavioural manifestation of self objectification, and as such it is measured independently from other facets of the original self objectification construct (i.e., body shame and control beliefs), but also used synonymously (Moradi & Huang, 2008; Tiggemann, 2013). It is important to note that body surveillance and IAc (or other aspects of interoception) are different constructs; the former includes an external perspective and evaluation, whereas the latter refers to internal body related sensations. Research has indicated a relationship between body surveillance and negative body image or distortion in both clinical (i.e., eating disorders, depression) and non-clinical samples (Calogero, Davis, & Thompson, 2005; Dakanalis, Timko, Clerici, Riva, & Carrà, 2017; Fitzsimmons-Craft et al., 2012; Moradi & Huang, 2008; Peat & Muehlenkamp, 2011; Tiggemann & Kuring, 2004). Self objectification is proposed to predict body image problems, and body surveillance has mediated the relationship between internalised thin ideals and body image dissatisfaction in previous research (Fitzsimmons-Craft et al., 2012; Fredrickson & Roberts, 1997; Knauss, Paxton, & Alsaker, 2008; Myers & Crowther, 2007; Tiggemann & Williams, 2012). More recently, Fitzsimmons-Craft and colleagues (2015) found that a higher level of body surveillance was moderately associated with increased body dissatisfaction. Other research suggests that less external body orientation is important for maintaining a positive body image (Avalos & Tylka, 2006; Homan & Tylka, 2014).

We suggest that Objectification Theory (Fredrickson & Roberts, 1997) may provide insight into previous findings that individuals with diminished IAc express higher body image dissatisfaction; while those with improved IAc demonstrate a more positive body image (Duschek et al., 2015; Emanuelsen et al., 2015). We believe that diminished accuracy in perceiving one’s internal signals may lead a person to rely on external sources of information concerning the bodily self, or vice versa.

Miller and colleagues (1981, p. 404) define public body consciousness, another concept of external orientation concerning one's appearance, as “a chronic tendency to focus on and be concerned with the external appearance of the body”. Individuals who are high in public body consciousness typically view themselves from an outsider's perspective, monitoring their appearance and behaviour to facilitate social interaction (Ainley & Tsakiris, 2013; Miller et al., 1981). Although distinct constructs, one could argue that public body consciousness is closely related to body surveillance, insofar as both constructs concern viewing oneself as a social object, an external orientation for information concerning one's body, and a preoccupation with appearance (Miner-Rubino, Twenge, & Fredrickson, 2002). Body surveillance, however, primarily differs from public body consciousness in that the individual takes on the perspective of the observer, as opposed to merely being aware of it (Miller et al., 1981; Miner-Rubino et al., 2002). In this way, it is likely a more disembodied experience than the awareness of self from a public perspective (Miner-Rubino et al., 2002).

Our aim was to investigate how internal orientation (i.e., interoception) and external orientation (i.e., public body consciousness and body surveillance) influence body image dissatisfaction. Research investigating similar associations (Ainley & Tsakiris, 2013; Duschek et al., 2015; Emanuelsen et al., 2015) has not included these constructs in one empirical study. Additionally, as this previous work investigated cross-sectional data, spontaneous fluctuation cannot be excluded; thus, we have used a short-term longitudinal study to explore their relation.

Based on the aforementioned theoretical considerations and empirical findings, low level of IAc and the proclivity to assess one's body from an outsider's perspective should predict a negative body image. In the present research, we expected that IAc, body surveillance and public body consciousness at baseline (t1) would predict body image dissatisfaction 8 weeks later (t2) (Hypothesis 1). We also considered the possibility that low IAc and high levels of body image dissatisfaction may increase the tendency to view oneself from a third party perspective. Therefore, alternately, we expected that IAc and body image dissatisfaction at t1 would predict body surveillance at t2 (Hypothesis 2).

## Material and Method

### Participants

Assuming α = .05, 1-β = .80, and a medium effect size (.15; in the lack of empirical data, this estimation was based on theoretical considerations), the minimum sample size for a multiple linear regression analysis with 6 predictor variables is 97 (Faul, Erdfelder, Lang, & Buchner, 2007). Participants in this research were Norwegian (*n* = 59, 74.6% female, 24.8 ± 5.09 yrs) and Hungarian (*n* = 38, 65.8% female, 21.3 ± 1.60 yrs) students enrolled at a University in Hungary. Norwegian students were enrolled in an English language international program. The research was approved by the Research Ethics Committee of the institution. Participation was voluntary, and all participants signed an informed consent form before the measurements. The English versions of the questionnaires were administered for the Norwegian students and the Hungarian version for the Hungarian participants.

### Measures

#### The Body Surveillance Subscale of Objectified Body Consciousness Scale

The scale was developed by McKinley and Hyde (1996) to assess negative body experience from a social constructionist point of view. The questionnaire measures the experience of the body as an object to be viewed by others and the beliefs underlying this experience. For the purpose of this research, we have chosen to use only the 8 item body surveillance subscale, which uses a 7 point Likert-scale. Higher values indicate higher surveillance tendency. Cronbach's alpha in the present study was .75 at t1, and .70 at t2.

#### The Public Body Consciousness Scale

The scale was developed by Miller and colleagues (1981) as part of the Body Consciousness Questionnaire, and it consists of 5 items rated on a 5 point Likert-scale. Higher scores indicate more importance placed on individual appearance. Cronbach's alpha for the public body consciousness subscale in the present study was .71 at t1.

#### The Body Image Ideals Questionnaire

The questionnaire was developed by Cash and Szymanski (1995) to provide a reliable assessment of participants' evaluation of their own physical appearance, and asks two questions with regard to each of 11 physical characteristics, including muscle tone, hair texture, complexion and various physical abilities (e.g., coordination, strength). Responses are indicated on a 4 point Likert-type scale. The first question asks participants to what extent they feel that they match their physical ideals; the second question asks how important it is to the participant that their actual attributes match their ideals. Higher scores on the scale indicate a greater overall level of body image dissatisfaction. In the current study, the internal consistency of the questionnaire was .65 at t1, and .69 at t2.

#### The Mental Heartbeat Tracking Method

Interoceptive accuracy was assessed using the mental tracking method (Schandry, 1981), a widely used paradigm. In healthy individuals, there is a correspondence between the performance on the Schandry-task and the mean amplitude of heartbeat evoked potential (Pollatos & Schandry, 2004), an EEG potential associated with the heartbeat, which is also higher during the Schandry-task than during periods of rest (Schulz et al., 2015). During the task, participants were asked to count their perceived heartbeats silently. They were not allowed to monitor their pulse (e.g., palpating the wrist or neck artery) or use any other physical techniques that might help them to count more accurately. They were further instructed to count uncertain sensations but to refrain from guessing. Upon hearing a “start” cue, participants began to silently count their own heartbeats until a “stop” cue was given, at which point they reported the number of heartbeats counted to an experimenter. At the same time, the experimenter counted and recorded the participants’ heartbeats using a Polar watch (model RS-400) with a chest strap. This procedure was administered for one 15 second warm-up trial followed by three subsequent intervals (30, 40, and 100 seconds) presented in random order, with a 10 second break in between. Following the initial trial, participants were asked to indicate how they arrived at the reported number of heartbeats. Subjects who reported guessing or counting seconds were encouraged to count only the perceived heartbeats for the remaining three trials. The experimenter explained that accuracy is regarded as neither positive nor negative. Subjects were not aware of the length of the intervals and no feedback about performance was given. IAc is the mean score of the formula: 1 – [(|recorded heartbeats – counted heartbeats|)/recorded heartbeats] calculated for each of the three time trials. Cronbach’s alpha coefficient for the index was .924.

### Procedure

Participants filled out an on-line test battery one day prior to a scheduled meeting with the experimenter (t1). At the meeting, participants were seated in a quiet room. After a brief introduction to the mental tracking method, participants were asked to relax, breath normally, and focus on the beating of their heart. Participants completed the on-line self report battery a second time 8 weeks later (t2). This period of time appears long enough to capture short-term fluctuations and fits within a typical 12-week university semester, while avoiding the inclusion of the stressful first and final 2 weeks of the semester.

This research was part of a larger study, thus participants took part in other measurements as well. Concerning the variables used in the present paper, only baseline interoceptive accuracy values were included in another research (Ferentzi, Drew, Tihanyi, & Köteles, 2018).

### Statistical Analysis

Statistical analysis was conducted using the JASP v0.8.5.1 software (JASP Team, 2019). Based on the results of normality analysis (Shapiro-Wilk tests), parametric statistical methods were used throughout the analysis. Differences between groups with respect to age and sex were checked using Student *t*-test and chi-square test, respectively. Concerning the assessed psychological variables, the two national groups were compared using Student *t*-test. Cross-sectional associations among variables at t1 were checked using Pearson correlation. Longitudinal associations (Hypothesis 1 and 2) were investigated using multiple linear regression analysis. In Step 1 the baseline value of the respective criterion variable was entered; in Step 2, group affiliation (Hungarian = 1; Norwegian = 2), gender (male = 1; female = 2), t1 values of interoceptive accuracy, body surveillance, and (only for body image dissatisfaction) public body consciousness were stepped in.

## Results

Descriptive statistics, group level comparisons, and baseline correlations are presented in Table 1 and Table 2, respectively. A statistically significant difference between groups in age, *t*(95) = -4.104, *p* < .001, *d* = -0.854, but not in sex ratio, χ^2^(1) = 0.869, *p* = .351, was found. The two groups showed significant differences with respect to body surveillance and public body consciousness at t1, and body image dissatisfaction at t2. Concerning baseline measures, a significant negative medium level association between body surveillance and IAc was found in the Hungarian group. In the Norwegian group, body surveillance was moderately associated with body image dissatisfaction and weakly with public body consciousness, and public body consciousness was negatively associated with IAc (for details, see Table 2).

**Table 1:** Descriptive Statistics (Mean ± Standard Deviation) of the Assessed Variables Split by Group, and Results of Student t-Tests Comparing the Two Groups

Variable	Hungarians *n = 38*	Norwegians *n = 59*	*t*(95)	*p*	Cohen‘s *d*
Body surveillance at t1	36.04 ± 5.174	33.05 ± 7.454	2.159	.033	0.449
Body surveillance at t2	33.68 ± 5.132	32.03 ± 6.465	1.322	.189	0.275
Body image dissatisfaction at t1	1.58 ± 1.327	1.41 ± .883	0.757	.451	0.157
Body image dissatisfaction at t2	1.96 ± 1.292	1.33 ± .797	3.015	.003	0.627
Public body consciousness at t1	24.16 ± 2.937	21.00 ± 3.634	4.492	< .001	0.934
IAc at t1	.46 ± .249	.52 ± .269	-1.087	.280	-0.226

*Note.* IAc = Interoceptive accuracy; t1 = Baseline; t2 = 8 weeks later.

**Table 2:** Pearson’s Correlations Among Variables at Baseline

Variable	1	2	3	4
1. Body image dissatisfaction	-	.14	-.07	.11
2. Body surveillance	.44**	–	.18	-.40*
3. Public body consciousness	.11	.32*	–	-.18
4. IAc	-.11	-.11	-.33*	–

*Note.* Upper triangle = Hungarians (*n* = 38); Lower triangle = Norwegians (*n* = 59); IAc = interoceptive accuracy.

*p < .05. **p < .01.

In the multiple linear regression analysis predicting body image dissatisfaction at t2 (Hypothesis 1), baseline BIQ score explained 23.1% of the total variance (*p* < .001) in Step 1. In Step 2, the regression equation explained 32.6% of the total variance (*p* < .001). Predictors of body image dissatisfaction at t2 were baseline body image dissatisfaction, group, and gender (*p* < .1), but not IAc, body surveillance, and public body consciousness (for details, see Table 3). Group association was negative, while gender had a positive association; thus, all other factors being equal, Hungarian nationality and female gender predicted higher levels of body image dissatisfaction at t2.

**Table 3:** Results of the Multiple Linear Regression With Body Image Dissatisfaction at t2 as the Dependent Variable

Step	*B*	*SE_B_*	β	95% CI (*LL*, *UL*)	*p*	Zero-order correlation	Partial correlation
Step 1: *R*^2^ = .231, *p* < .001							
Body image dissatisfaction at t1	0.474	0.089	0.481	0.298, 0.650	< .001	.481	.481
Step 2: *R*^2^ = .326, *p* < .001							
Body image dissatisfaction at t1	0.439	0.091	0.446	0.259, 0.619	< .001	.481	.455
Group	-0.622	0.210	-0.288	-1.038, -0.205	0.004	-.296	-.298
Gender	0.371	0.214	0.159	-0.055, 0.797	0.087	.202	.179
IAc at t1	-0.147	0.382	-0.036	-0.905, 0.611	0.701	-.100	-.041
Body surveillance at t1	-0.005	0.015	-0.035	-0.036, 0.025	0.725	.191	-.037
Public body consciousness at t1	-0.006	0.029	-0.019	-0.064, 0.053	0.851	.140	-.020

*Note.* IAc = Interoceptive accuracy; t1 = Baseline; t2 = 8 weeks later.

The regression equation predicting body surveillance at t2 (Hypothesis 2) explained 49.7% of total variance in Step 1, and 51.7% in Step 2. The only significant predictor was baseline body surveillance score (for details, see Table 4).

**Table 4:** Results of the Multiple Linear Regression With Body Surveillance at t2 as Dependent Variable

Step	*B*	*SE_B_*	β	95% CI (*LL*, *UL*)	*p*	Zero-order correlation	Partial correlation
Step 1: *R*^2^ = .497, *p* < .001							
Body surveillance at t1	0.624	0.064	0.705	0.496, 0.752	< .001	.705	.705
Step 2: *R*^2^ = .517, *p* < .001							
Body surveillance at t1	0.640	0.071	0.724	0.499, 0.781	< .001	.705	.687
Group	-0.069	0.927	-0.006	-1.911, 1.773	.941	-.134	-0.008
Gender	1.571	1.021	0.119	-0.458, 3.600	.128	.206	.159
IAc at t1	2.468	1.770	0.108	-1.049, 5.985	.167	-.073	.145
Body image dissatisfaction at t1	-0.327	0.431	-0.059	-1.184, 0.529	.450	.179	-.079

*Note.* IAc = Interoceptive accuracy; T1 = Baseline; T2 = 8 weeks later.

## Discussion

This study investigated the temporal relationships among the external orientation toward the body, body image, and interoceptive accuracy (heartbeat tracking ability) assessing a general student sample of young Hungarians and Norwegians. Dissatisfaction with body image 8 weeks later was predicted by baseline dissatisfaction, Hungarian nationality, and female gender, but not by interoceptive accuracy or external body orientation. Body surveillance was predicted only by baseline body surveillance but not by gender, nationality, interoceptive accuracy, or dissatisfaction with body image.

Based on past research, we expected that interoceptive accuracy (IAc) and constructs representing an external body orientation (public body consciousness and body surveillance) would predict body image dissatisfaction (Ainley & Tsakiris, 2013; Emanuelsen et al., 2015; Fitzsimmons-Craft et al., 2012; Fredrickson & Roberts, 1997). Whereas these hypotheses were not supported by our data, nationality and gender were predictors of change. Nationality related findings are difficult to explain as (1) the size of the two samples was not equal and (2) there was a significant difference between the two groups with respect to age. Generally, Hungarian adolescents experience higher levels of body concerns when compared to other European nationalities (Papp, Urbán, Czeglédi, Babusa, & Túry, 2013); this tendency, along with their younger age, might have made the temporal fluctuations of body image dissatisfaction more marked.

The result that female gender predicted (although only at a trend level) greater change in body image dissatisfaction was not surprising, as empirical evidence shows that females generally experience higher levels of body image dissatisfaction (Grabe, Ward, & Hyde, 2008; Grogan, 2016; Tiggemann, 2004). In past studies, both Norwegian and Hungarian adolescent females have shown higher levels of body image dissatisfaction than their male counterparts (Meland, Haugland, & Breidablik, 2007; Papp et al., 2013). Even at very young ages, girls are more likely to exhibit body concerns, and be more dissatisfied with their bodies (Grogan, 2016), perpetuated by the internalised ideals promoted by modern western culture and media (Grabe et al., 2008; Myers & Crowther, 2007).

Although a previous cross-sectional study revealed a medium level reverse correlation between IAc and self objectification (Ainley & Tsakiris, 2013), this was replicated only in the Hungarian sample in the present study. Moreover, results of our second regression model indicate that IAc does not explain variance in changes of body surveillance. According to our results, the only predictor of self objectification at t2 was the baseline self objectification score; the strong association between the two (β = 0.705) indicates high temporal stability. Therefore, temporal associations with IAc are difficult to detect if the effect size of IAc is low and additional factors are controlled for. Temporal stability of body image dissatisfaction was lower (β = 0.481); still, low to medium level associations between body image dissatisfaction and IAc reported in cross-sectional studies (Duschek et al., 2015; Emanuelsen et al., 2015) might have been too weak to detect in the current study. Moreover, the instruction used in the Schandry-task was more strict than usual with respect to allowing estimation. This might have resulted in lower IAc scores with less variance than in other studies and possibly influenced participants’ response tendencies (for more detail, see the limitations section). Overall, the lack of predictive associations among self-objectification, body image dissatisfaction, and IAc indicates that the ability to accurately sense our bodily signals is not among the significant factors that influence how we monitor and envision our body’s appearance. For example, self-reported (i.e., conscious) aspects of interoception (e.g., interoceptive sensibility and body awareness) might play a more important role in these processes. Additionally, as Tiggemann (2004) has pointed out, body surveillance and body image dissatisfaction do not necessarily go hand-in-hand, especially considering the multi-faceted complexity of the body image dissatisfaction construct and individual differences in the internalised-ideal used as a comparator between the perceived body and ideal body.

The current study is not without limitations. First, an 8 week time frame might not be sufficient to reveal on complex time-related associations among multiple variables. Second, internal consistency for the Body Image Ideals Questionnaire and public body consciousness scores were acceptable but low when compared to previous research (Ainley & Tsakiris, 2013; Dakanalis et al., 2017; Miller et al., 1981). Third, although widely accepted, the mental tracking paradigm (Schandry, 1981) has received some criticism for the potential that participant responses could be influenced by previous knowledge about their own heart rate, or expectations concerning their ability to detect their heart rate accurately (Ring, Brener, Knapp, & Mailloux, 2015). Instruction given to participants can also bias the measurement (Desmedt, Luminet, & Corneille, 2018). For example, participants with the tendency to please others may produce lower overall IAc scores when given strict instructions that do not allow guessing, but produce inflated IAc scores when given permissive instructions that do allow for guessing. Individuals with a desire to please others could also be likely to aspire to social norms and experience body image dissatisfaction, which may provide some explanation for why we did not find an expected relationship between increased body image dissatisfaction and lower IAc at baseline. Finally, although Norwegian students in the present research have a high level of proficiency and study in English on a daily basis, the questionnaires were neither translated into Norwegian nor validated for use with a Norwegian population. There may be subtle variations in responses due to second language understanding.

In summary, culture and gender differences should be considered when designing interventions for improving body image, as subgroups of the population may show disparate patterns of association among related constructs.
